# Neglected role of microelements in determining soil microbial communities and fruit micronutrients in loquat orchards

**DOI:** 10.3389/fmicb.2024.1447921

**Published:** 2024-08-21

**Authors:** Xianting Wang, Li Wang, Bibo Wu, Zhaofeng Yuan, Yingying Zhong, Lin Qi, Miao Wang, Yuping Wu, Tida Ge, Zhenke Zhu

**Affiliations:** ^1^Yinzhou Station of Agricultural Technical Extension, Ningbo, China; ^2^State Key Laboratory for Managing Biotic and Chemical Threats to the Quality and Safety of Agro-products, Institute of Plant Virology, Ningbo University, Ningbo, China; ^3^Ninghai County Forestry Specialty Technology Promotion Station, Ningbo, China; ^4^Ningbo Customs Technology Center, Ningbo, China; ^5^Ningbo Agricultural and Rural Green Development Center, Ningbo, China

**Keywords:** bacteria, fungi, protists, microelement, community assembly

## Abstract

**Introduction:**

The relationships among microelements and soil microbial communities are essential for understanding the maintenance of soil's ecological functions and their effects on fruit quality in orchards. However, these relationships have not been adequately studied, despite the importance of microelements for the growth of microorganisms and plants.

**Methods:**

To address this research gap, we investigated the relationships among microelements (K, Ca, Na, Mg, Fe, Mn, Zn, and Cu), the diversity and composition of soil microbiomes, and fruit quality in loquat orchards.

**Results:**

We found that microelements explained more variations in microbial community structures than geographic position, basic soil properties, and macroelements, with 19.6–42.6% of bacterial, 4.3–27.7% of fungal, and 5.9–18.8% of protistan genera significantly correlated with microelements. Among the microelements, AMg and ACu were the most influential in determining the soil microbiome. The soil microbes exhibited varied threshold values for environmental breadth among the microelements, with the broadest range for AMg and the narrowest for AZn. Additionally, the microbes showed significant phylogenetic signals for all microelements, with an increasing divergence of soil microelements. The dominant community assembly shifted from homogeneous selection to stochastic, and then to heterogeneous selection. Moreover, microelements and the microbiome were the top two factors individually explaining 11.0 and 11.4% of fruit quality variation, respectively.

**Discussion:**

These results highlight the importance of microelement fertilization in orchard management and provide scientific guidance for improving fruit quality.

## Highlights

- Microelements and microbiomes mainly affect fruit quality in loquat orchards.
- Microbial community structures are explained primarily by microelements.
- Microbes demonstrated significant phylogenetic signals for all microelements.

## Introduction

Soil microorganisms are the most diverse taxonomic and functional groups in soil and are essential for maintaining soil ecological functions (Wu et al., 2024). They promote the soil nutrient availability (Cao et al., 2023) and help plants resist pathogens (Trivedi et al., 2017) and stress (Zhao et al., 2024), thereby affecting plant growth, health, and yields. Soil microorganisms can also influence the growth of fruit trees, impacting fruit quality and quantity. For example, Chen et al. (2023) claimed that the rhizospheric microbiome may enhance the production of fruit chemical components in *Cinnamomum migao* H. W. Li. In addition, Su et al. (2023) reported that rhizospheric and endophytic microorganisms promote monoterpenes production in citrus by providing their precursors. In our previous study, we found that the richness of soil bacteria and protists positively correlated with the soil multi-element cycle index and average fruit weight of the loquat tree (Wang et al., 2024). This result indicates that soil microbial communities can boost fruit yields by facilitating nutrient provision in soils.

Due to the important functions of soil microorganisms, several studies have focused on the effects of various environmental factors on soil microorganisms, such as climate (Sáez-Sandino et al., 2023), geographical distance (Liu et al., 2019), and soil physical and chemical factors (Zhang et al., 2021; Tang et al., 2023; Zhong et al., 2023). However, regarding physical and chemical factors of soils, researchers have largely focused on soil pH, electrical conductivity (EC), moisture, and macroelements such as carbon, nitrogen, and phosphorus (Zhang et al., 2021, 2023; Tang et al., 2023; Zhong et al., 2023). Microelements (K, Ca, Na, Mg, Fe, Mn, Zn, and Cu) also significantly affect the growth and activity of microorganisms (Peng et al., 2022; Dai et al., 2023). The interactions between microbiome and micronutrients are critical for the regulating of plant health (Noman et al., 2024). Microelements often act as cofactors or provide structural support for various enzymes, thus affecting microbial metabolism (Welch and Shuman, 1995; Fischer et al., 2015; Feng et al., 2019). In every enzyme class, up to 36% of proteins require microelements such as K, Na, Ca, Mg, Fe, Mn, Zn, and Cu to maintain enzymatic functions (Waldron et al., 2009; Murdoch and Skaar, 2022). The homeostasis of Na^+^ and K^+^ is essential for microbial survival and pivotally influences osmotic pressure regulation, pH homeostasis, protein synthesis regulation, membrane potential regulation, and electrical signal conduction (Rath and Rousk, 2015; Armstrong and Hollingworth, 2021; Shu and Huang, 2022). Ca^2+^ affects microbial responses to temperature, salt, osmotic stress, and pressure (Shu and Huang, 2022) and can impact soil microbial biomass, soil respiration, and organic matter conversion (Wong et al., 2008; Shabtai et al., 2023). Fe and Mn are prominent electron acceptors in microbial respiration (Dubinsky et al., 2010; Whalen et al., 2018).

In orchards, micronutrients may play even more important roles because they determine the mineral and elemental contents in fruits, crucially influencing their nutritional value. However, few studies have focused on the effects of microelements on microbial composition and function in orchards. Here, we collected loquat fruit and paired soil samples around trees from Ninghai County, Zhejiang Province, China, which is the origin of superior varieties of the “Ninghai Bai” loquat. We measured soil basic physicochemical properties, macroelement and microelement content, soil microbial communities, and fruit quality to examine the impacts of microelements on soil microbial communities and loquat fruit quality.

## Materials and methods

### Sample collection

In this study, we randomly selected 10 loquat orchards from different geographical locations in Ninghai County, Zhejiang Province, China. Paired soil and fruit samples were collected from six randomly chosen loquat trees in each orchard (Supplementary Figure 1). Detailed information about the orchards is presented in Supplementary Table 1. Sampling took place during the loquat harvest period in August 2021, with the latitude, longitude, and altitude of each tree recorded at each sampling site. For each tree, 40 loquat fruits were gently placed into a plastic box. Additionally, nine bulk soil core samples were collected at a depth of 0–20 cm around each tree and mixed to form a composite soil sample. Soil and fruit samples were immediately transported to the laboratory on ice after collection.

### Fruit quality determination

In the laboratory, the fruit samples were divided into two parts: one part was used to determine the weight, soluble solids, and vitamin C content of fresh fruit, while the remaining part was freeze-dried and stored at −80°C to evaluate dietary fiber, reducing sugars, total flavones, and mineral elemental contents (K, Na, Ca, Mg, Fe, Zn, Cu, Co, Mo, Mn, Pb, B, Ba, Tl, Li, and Ni). Detailed information on the methods used for fruit quality determination is provided in our previous paper Wang et al. (2024) and in the Supplementary material.

### Soil physicochemical properties, macroelements, and microelements

The methods for determining the soil physicochemical properties, including pH, electrical conductivity (EC), moisture, and macroelemental contents [soil organic carbon (SOC), dissolved organic carbon (DOC), dissolved organic nitrogen (DON), nitrate (${\text{NO}}_{3}^{-}$), ammonia (${\text{NH}}_{4}^{+}$), and available phosphorus (AP)], have been described in our previous paper Wang et al. (2024). The concentrations of microelements, including available K, Ca, Na, Mg, Fe, Mn, Zn, and Cu, were analyzed by atomic absorption spectrometry (AAS; Varian SpectrAA 220, SpectraLab Scientific, Inc., Canada).

### Total DNA extraction, amplicon sequencing, and bioinformatic analysis

In this study, we used the PowerSoil DNA extraction kit (Qiagen, Hilden, Germany) to extract total soil DNA. The target fragments of bacteria, fungi, and protists were amplified using the primers 16S rRNA 515F/907R, ITS5-1737F/ITS2-2043R, and TAReuk454FWD1/TAReukREV3, respectively (Supplementary Table 2; Du et al., 2022; Jiao et al., 2022). The raw sequences obtained from Illumina MiSeq PE300 were purified and analyzed using QIIME v.2.1.0 to obtain high-quality sequences (q > 20; Wang et al., 2023). Amplicon sequence variants (ASVs, 100% similarity) were designated using Deblur, and taxonomic classification was conducted by comparing the representative sequences to the SILVA version 138 reference, UNITE v8.0, and PR2 for bacterial, fungal, and protistan communities, respectively. To ensure uniform sampling depth, the ASV table was refined to 44,357, 70,730, and 62,305 sequences for bacteria, fungi, and protists in each sample before statistical analysis.

### Statistical analysis

The relationships between multiple environmental factors and soil bacterial, fungal, and protistan communities were explored using redundancy analysis (RDA) and Mantel tests. The effect of geographical location on community composition was estimated using permutational multivariate analysis of variance (PERMANOVA). Variance partitioning analysis (VPA) was used to investigate the relative contributions of geographic position, basic soil properties (pH, EC, moisture), macroelements, and microelements to the soil microbial communities. Correlations between environmental factors and dominant bacteria, fungi, and protists were determined by Spearman's correlation analysis at the phylum and genus levels. Bacterial, fungal, and protistan genera with the highest positive or negative correlation coefficients with microelements were selected for further linear regression analysis.

Threshold indicator taxa analysis was performed using the “TITAN2” package in R to evaluate the environmental thresholds of bacterial, fungal, and protistan taxa to various environmental factors. High environmental threshold values of microbes indicated their ability to adapt to a wide range of environmental conditions (Wan et al., 2021). The phylogenetic signals of bacterial, fungal, and protistan taxa to different environmental factors were evaluated via the Fritz-Purvis D-test using the “caper” package in R (Orme, 2013). The Fritz-Purvis D-test calculates the phylogenetic dispersion value (D) by comparing observed differences in sister branches on an environmental factor with expected differences from random phylogenetic patterns (Goberna and Verdú, 2016). –D + 1 = 0 indicates no significant phylogenetic signal in response to a given environmental factor, whereas –D + 1 > 0 indicates that microbial responses to that environmental factor are more conserved than expected by chance.

The β-nearest taxon index (βNTI) was calculated to estimate the assembly processes of soil bacterial, fungal, and protistan communities based on a null model using the “picante” package (Stegen et al., 2013). |βNTI| < 2 implies that community assembly is driven by stochastic processes (i.e., random dispersal, drift, and speciation), whereas a |βNTI| > 2 implies that deterministic processes dominate community assembly. Specifically, a βNTI > 2 indicates more phylogenetic turnover than expected, associated with variable selection, while a βNTI < −2 indicates less phylogenetic turnover, associated with homogeneous selection.

Environmental dissimilarity of the basic soil properties (pH, EC, moisture), macroelements, and microelements was assessed using the Bray-Curtis distance. The correlation between community assembly (βNTI) and environmental dissimilarity was analyzed via linear regression. Overall relationships between fruit quality and basic soil properties (pH, EC, and moisture), macroelements, microelements, and soil microbiomes were assessed using RDA and VPA. Molecular ecological networks (MENs) for the soil microbiome were constructed using a Pearson's correlation coefficient of 0.61 based on previous studies (Deng et al., 2012, 2016). The correlation between specific fruit quality indicators and the relative abundance of soil microbes in each module was evaluated using Spearman's correlation analysis.

## Results

### Overall relationships between microbial community and environmental factors

The bacterial communities in these loquat orchards were dominanted by Proteobacteria (37%), Acidobacteriota (17%), Bacteroidota (9%), Actinobacteriota (7%), Chloroflexi (5%), Planctomycetota (5%), Verrucomicrobiota (5%), Firmicutes (3%), and Myxococcota (2%). The fungal communities were dominanted by Ascomycota (47%), Mortierellomycota (9%), Basidiomycota (3%), and Rozellomycota (2%). While the protistan communities consisted of Opisthokonta (52%), Rhizaria (19%), Alveolata (12%), Archaeplastida (3%), Stramenopiles (3%), and Amoebozoa (2%; Supplementary Figure 2).

**Figure 1:**
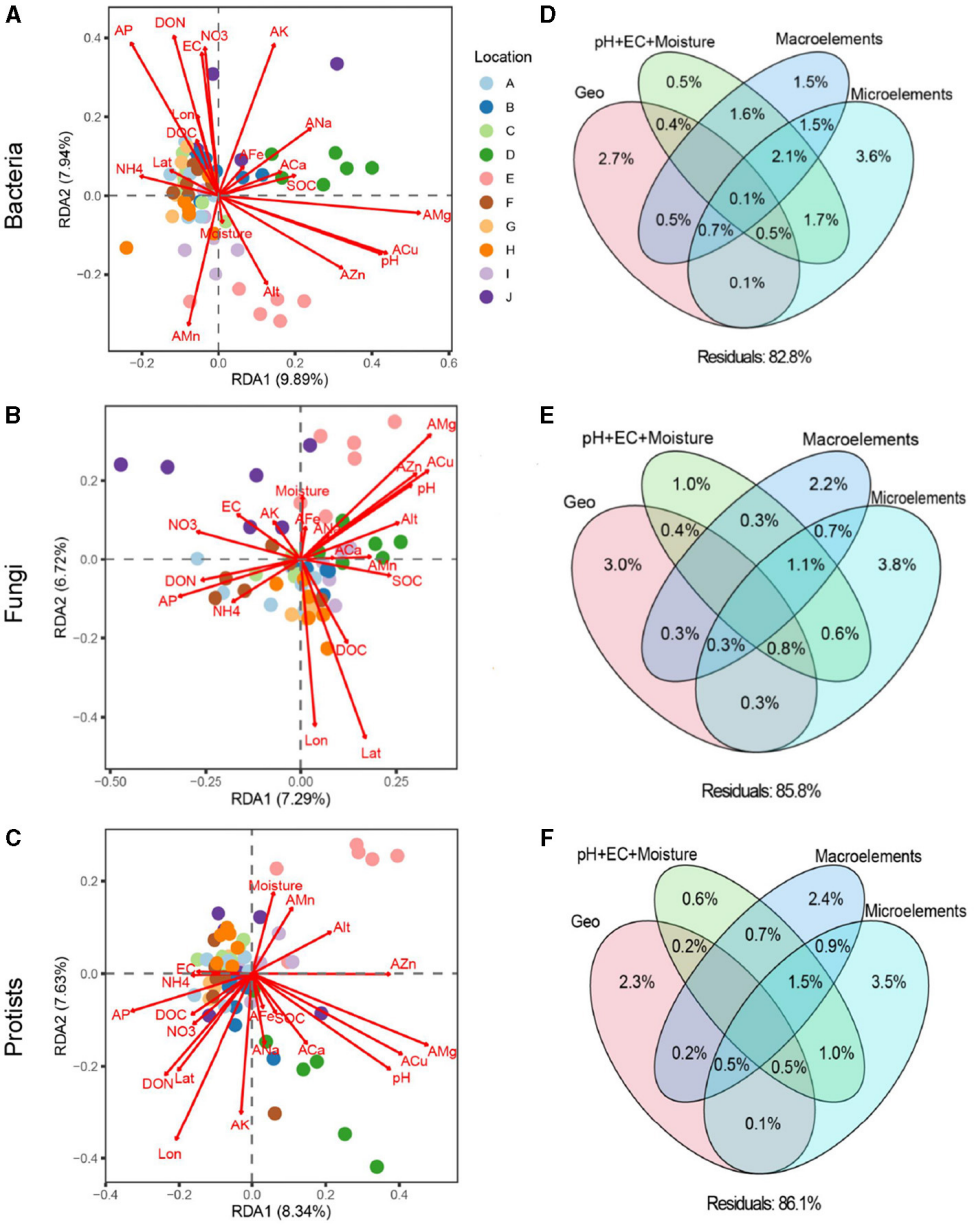
Redundancy analysis (RDA) between multiple environmental factors and soil bacterial **(A)**, fungal **(B)**, and protistan **(C)** communities. Variance decomposition analysis (VPA) investigating the relative contributions of geographic position (Longitude, latitude, and altitude), soil basic properties (pH+EC+moisture), macroelements (C, N, and P), and microelements (K, Ca, Na, Mg, Fe, Mn, Zn, and Cu) on soil bacterial **(D)**, fungal **(E)**, and protistan **(F)** communities.

PERMANOVA and RDA revealed that the soil bacterial (*F* = 7.86, *R*^2^ = 0.313, *P* < 0.001), fungal (*F* = 6.55, *R*^2^ = 0.263, *P* < 0.001), and protistan (*F* = 6.89, *R*^2^ = 0.288, *P* < 0.001) communities differed significantly among loquat orchards from different geographical locations (Figures 1A–C). Both the Mantel test and VPA indicated that soil microelements significantly and primarily determined soil microbial community structures compared to geographic position, basic soil properties, and macroelements (Figures 1D–F, Supplementary Table 3).

**Figure 2:**
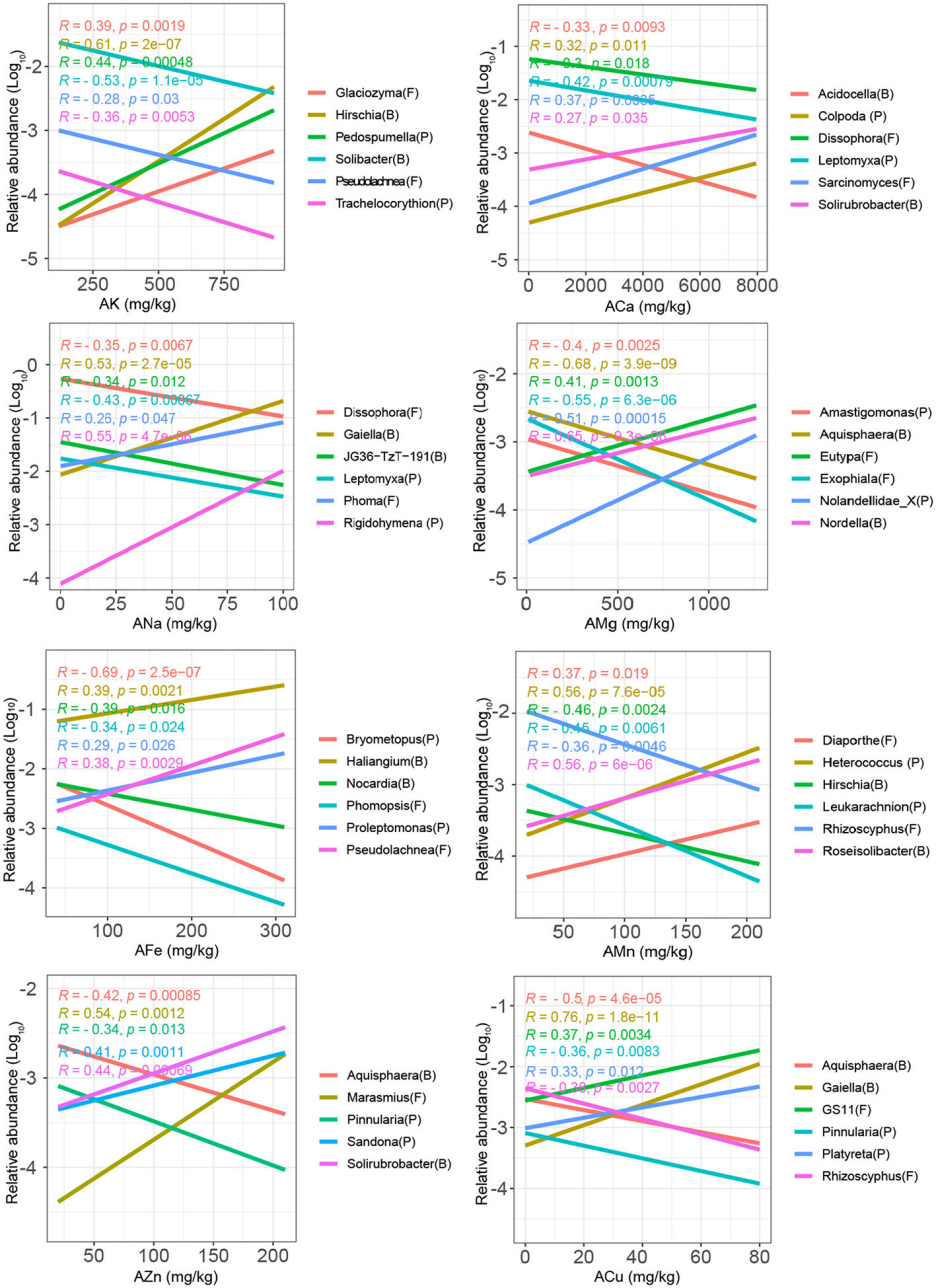
Linear regression between the relative abundances of soil microbial genera and microelements (K, Ca, Na, Mg, Fe, Mn, Zn, and Cu). The most significant (i.e., largest correlation coefficient) negatively and positively correlated genera with the microelements are presented in the figure.

The dominant role of microelements in shaping the soil microbial community structure was further confirmed through correlation analysis between the relative abundances of major microbial taxa and microelements (Figure 2 and Supplementary Figure 2, Table 1). At the phylum level, the relative abundances of *Myxococcus* and *Amoebozoa* were significantly positively correlated with AMg, whereas those of *Acidobacteriota, Planctomycetota*, and *Proteobacteria* were negatively correlated with AMg (Supplementary Figure 3, *P* < 0.05). Additionally, the relative abundances of *Myxococcota* and *Alveolata* were strongly correlated with ACu; *Bacteroidota* and *Firmicutes* were correlated with AK, *Acidobacteriota* were correlated with ANa, *Gemmatimonadota* were correlated with AMn, and *Myxococcota* were correlated with AFe (Supplementary Figure 3).

**Table 1:** Number of general where the relative abundance was significantly correlated with microelements.

**Microelements**	**Bacteria**		**Fungi**		**Protists**		**Total**
	(291)^a^		(47)^b^		(389)^c^		
	+^d^	-^e^	+	-	+	-	
AK	84	18	4	2	15	25	148
ANa	49	31	5	1	17	18	121
ACa	57	3	3	1	19	12	95
AMg	60	64	8	5	53	20	210
AFe	28	43	3	1	8	15	98
AMn	48	9	3	2	20	13	95
AZn	38	30	2	0	24	9	103
ACu	68	56	5	3	34	13	179

^a, b, c^Numbers in parentheses represent the total number of genera.

^d, e^+ and - represent positive and negative correlations, respectively.

At the genus level, 19.6–42.6% of bacteria, 4.3–27.7% of fungi, and 5.9–18.8% of protists were significantly positively or negatively correlated with microelements (Table 1). AMg and ACu had the highest numbers of correlated bacterial (124 and 124), fungal (13 and 8), and protistan (73 and 47) genera (Table 1). For example, *Nocardia, Phomopsis*, and *Bryometopus* had lower relative abundances, whereas *Haliangium, Pseudolachnea*, and *Proleptomonas* had higher relative abundances in soils with higher AMg concentrations (Figure 2). Similarly, *Aquisphaera, Rhizoscyphus*, and *Pinnularia* showed lower relative abundances, whereas *Gaiella, GS11*, and *Platyreta* showed higher relative abundances in soils with high ACu (Figure 2).

ACa, AMn, and AFe had the lowest numbers of correlated genera for the total microbial communities (95, 95, and 98, respectively; Table 1). Interestingly, for AMg, AZn, and ACu, the number of bacterial genera positively and negatively correlated with these microelements showed minimal differences (60 vs. 64, 38 vs. 30, and 68 vs. 56, respectively; Table 1). For AK, ACa, and AMn, the number of bacterial genera that were positively correlated with these microelements was obviously larger than that of the negatively correlated genera (84 vs. 18, 57 vs. 3, and 48 vs. 9, respectively; Table 1). These results suggest that most microorganisms consistently prefer high levels of AK, ACa, and AMn.

### Responses of microbial community assembly processes to microelements

**Figure 3:**
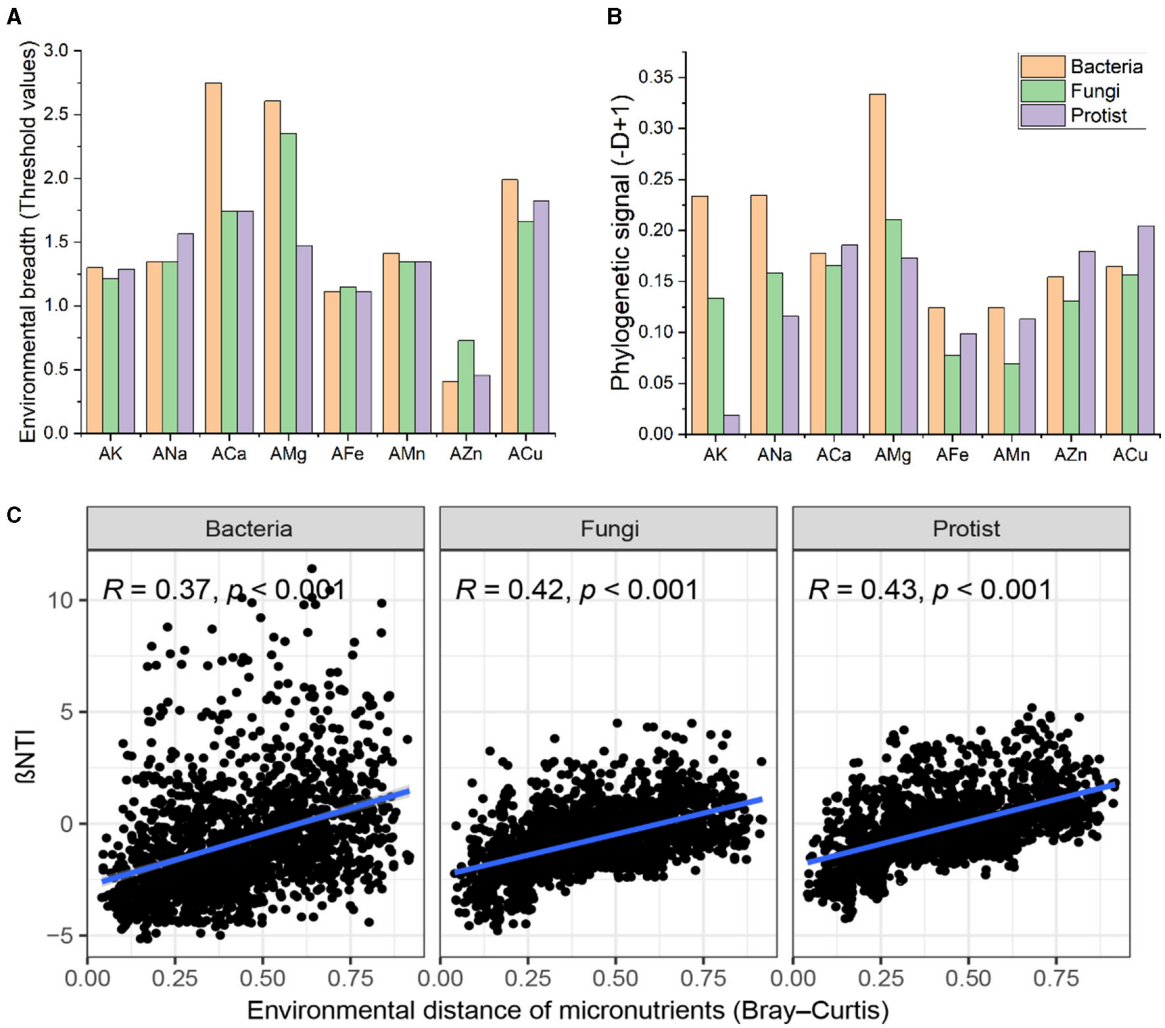
Environmental adaptation and community assembly of soil microbes. **(A)** Environmental breadth as estimated by the threshold values of soil bacteria, fungi, and protists in response to environmental variables, was calculated using threshold indicator taxa analysis. The threshold values were standardized using log10 (original threshold value + 1). **(B)** Phylogenetic signals showing trait conservatism for environmental preferences of soil bacterial, fungal, and protist communities using the D-test of Fritz and Purvis. **(C)** Relationships between NTI and differences in soil microelements in bacterial, fungal, and protistan communities.

Given the significant role of microelements in determining microbial community structure and composition, we further evaluated the environmental thresholds of bacterial, fungal, and protistan taxa in response to various environmental factors (Figure 3A). ANOVA revealed striking discrepancies in the threshold values for environmental breadth among different microelements (*F* = 8.961, *R*^2^ = 0.708, *P* < 0.001). However, there were no significant differences among soil bacteria, fungi, and protists (*F* = 0.461, *R*^2^ = 0.038, *P* = 0.665). This result indicates that the three taxa types had different adaptive ranges for different environmental factors but exhibited similar adaptive degrees to the same environmental factors. Soil microbes exhibited the broadest range of environmental thresholds for AMg and the narrowest range for AZn (Figure 3A).

Additionally, we examined the phylogenetic signals of soil bacteria, fungi, and protists to microelements using Fritz and Purvis's D-test (Figure 3B). This index could reflect the response characteristics of microorganisms to environmental factors, that was whether closely phylogenetically related microorganisms respond more similarly to environmental factors than the less phylogenetically related microorganisms. Soil bacteria, fungi, and protists showed significant phylogenetic signals for all microelements (Figure 3B). Moreover, to test the relationships between microelements and soil microbial communities, βNTI was calculate based on a null model. The results revealed that βNTI for the bacterial, fungal, and protistan communities was prominently positively correlated with dissimilarity in microelements (Figure 3C), implying a shift from homogeneous selection to stochasticity, and then to heterogeneous selection, in microbial community assembly along with increasing divergence of soil microelements. Overall, these results indicate that microelements crucially influence the assembly of microbial communities.

### Contributions of soils' abiotic properties and soil microbiomes to fruit quality

**Figure 4:**
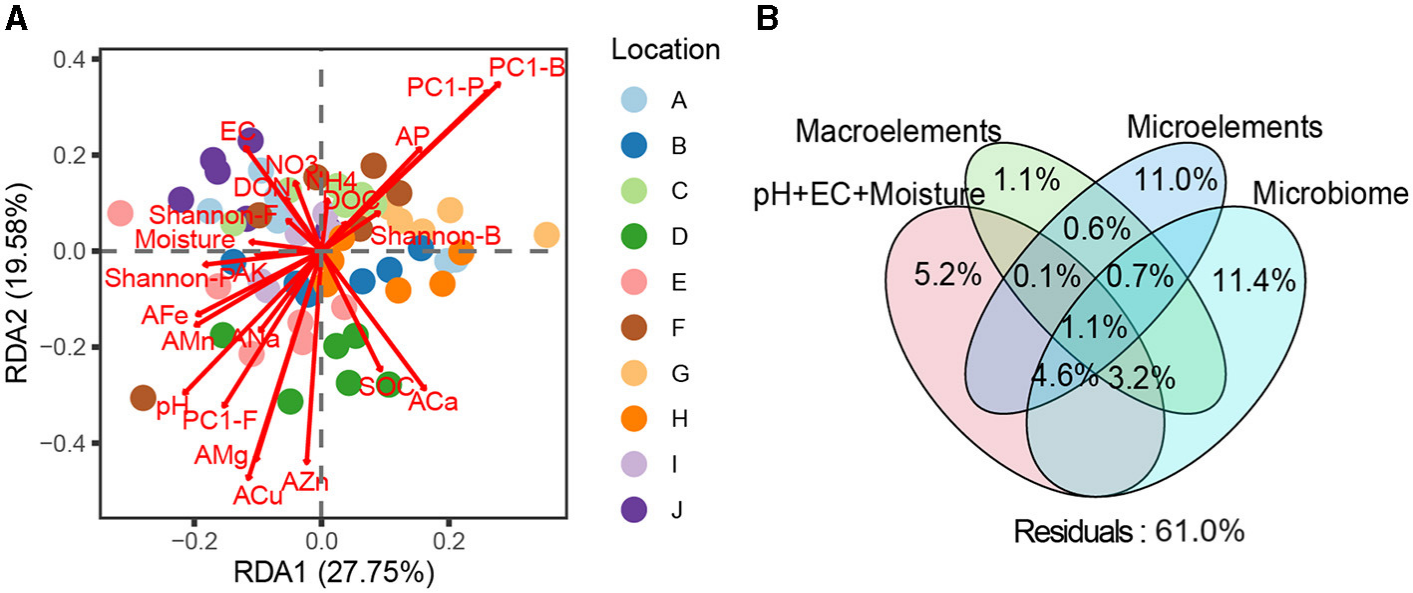
**(A)** Redundancy analysis (RDA) among multiple environmental factors, soil microbial community structure, and fruit quality of loquats. **(B)** Variance decomposition analysis (VPA) investigating the relative contributions of basic soil properties (pH+EC+moisture), macroelements (C, N, and P), microelements (K, Ca, Na, Mg, Fe, Mn, Zn, and Cu), and the soil microbiome to the fruit quality of loquats.

PERMANOVA and RDA revealed significant discrepancies in the quality of loquat fruits picked from different orchards (*F* = 6.32, *R*^2^ = 0.308, *P* < 0.001, Figure 4A). Basic soil properties, macroelements, microelements, and microbiome individually explained 5.2, 1.1, 11.0, and 11.4% of the variation in fruit quality, respectively (Figure 4B), suggesting that soil microelements and the microbiome were paramount factors influencing fruit quality. Furthermore, 3.2% of the variation in fruit quality was jointly explained by basic soil properties, macroelements, and the microbiome; 4.6% was jointly explained by basic soil properties, microelements, and the microbiome, and 1.1% was jointly explained by basic soil properties, macroelements, microelements, and the microbiome (Figure 4B). These results suggest that basic soil properties may affect fruit quality by regulating the soil microbiome or the availability of soil macro- and microelements.

**Figure 5:**
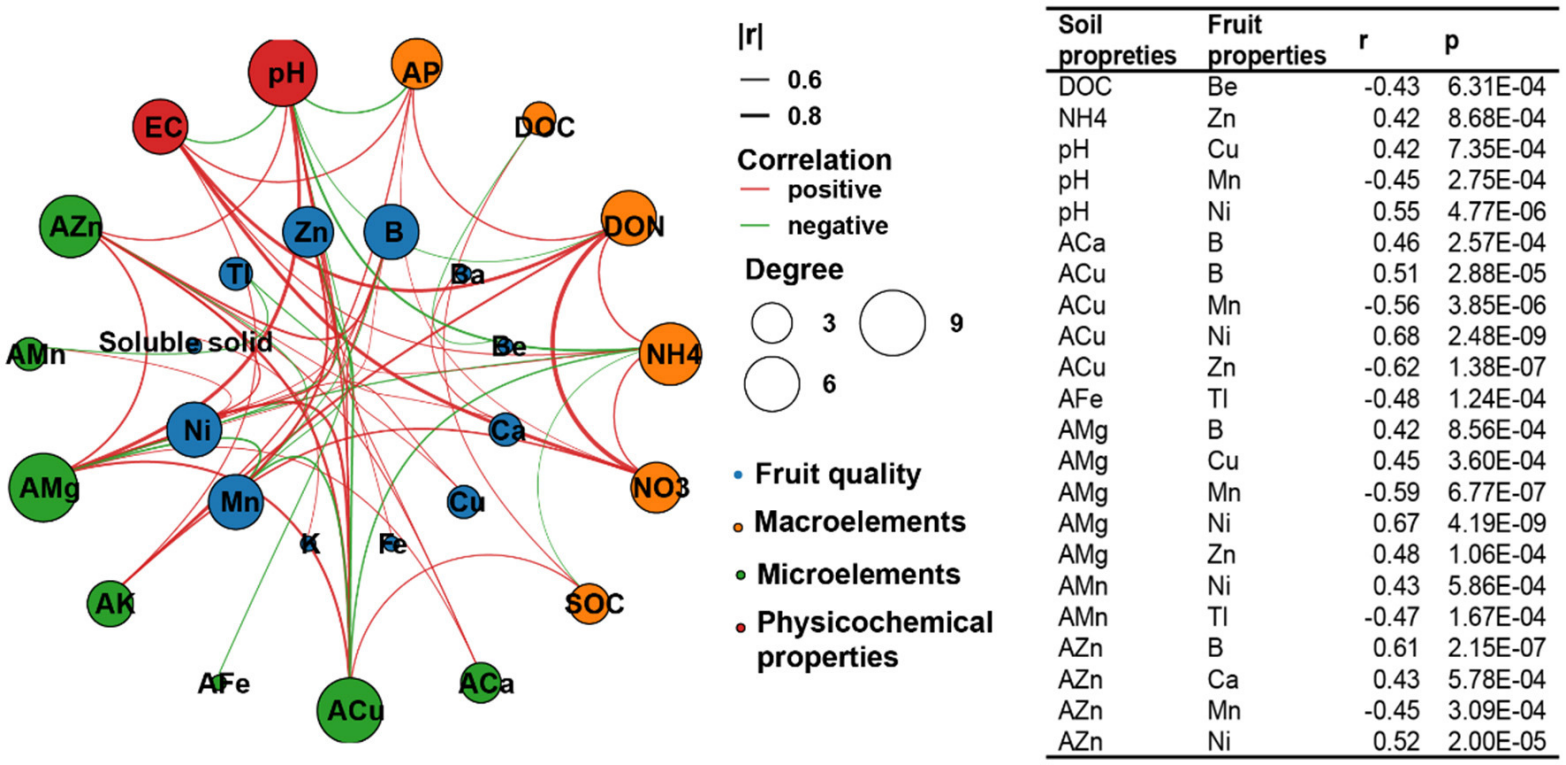
Network analysis of soil abiotic properties and loquat fruit quality based on Spearman's correlation coefficients.

**Figure 6:**
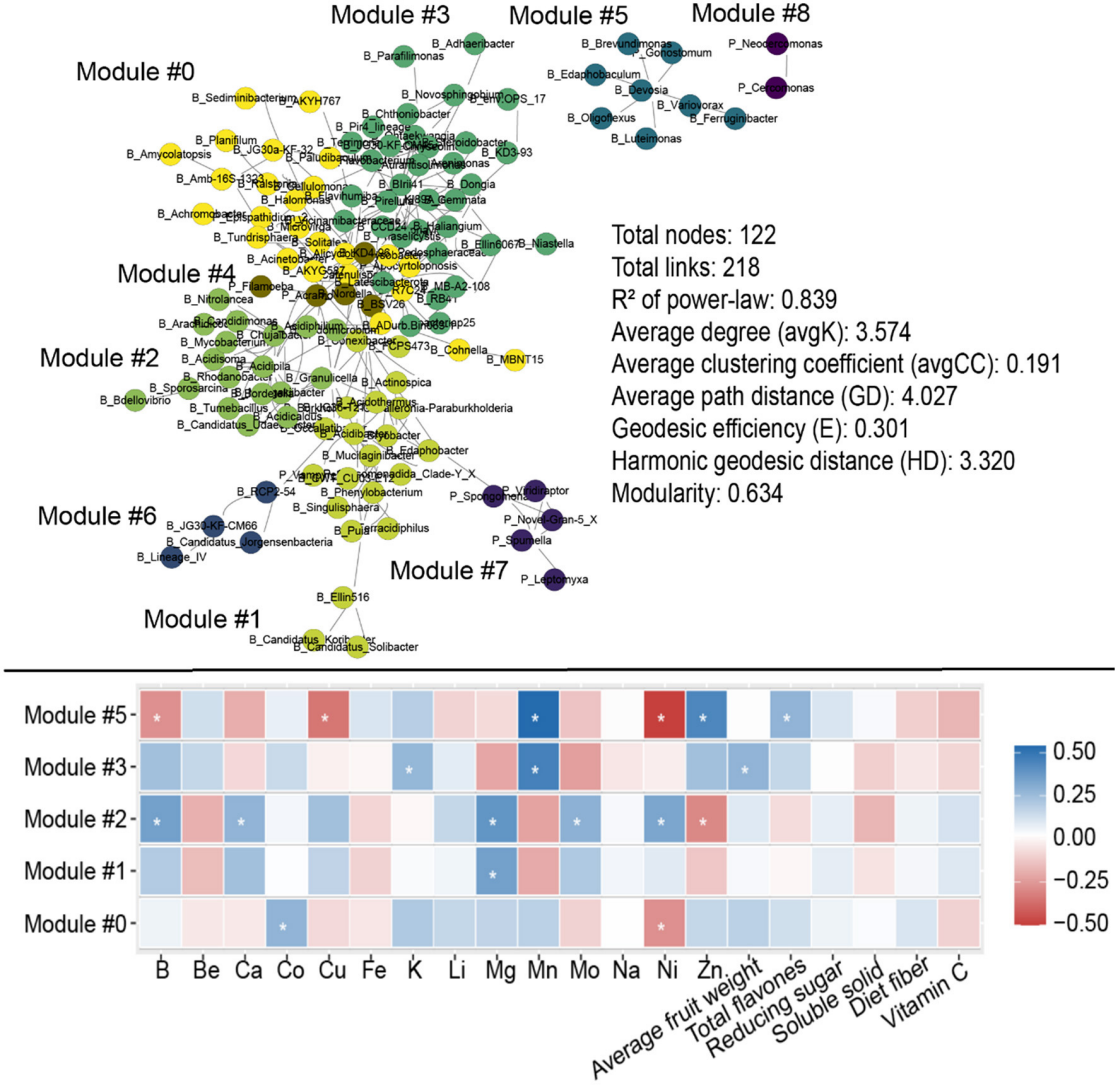
Molecular ecological networks (MENs) for soil microbial communities, and the correlation between specific fruit quality indicators and relative abundances of soil microbes in each module evaluated using Spearman's correlation analysis.

We then conducted a network analysis between soil abiotic properties and the quality of loquat fruits (Figure 5), as well as a correlation analysis between the relative abundance of soil microbes in the molecular ecological networks module and specific fruit quality indicators (Figure 6), to identify the specific abiotic properties and microorganisms influencing fruit quality. Soil abiotic properties were related to the mineral element contents in the fruit but not to the average fruit weight, soluble solids, vitamin C, total flavones, dietary fiber, and reducing sugars (Figure 5). Among the basic soil properties, pH was significantly correlated with Cu, Mn, and Ni in loquat fruits; for macroelements, DOC was negatively correlated with Be, and ${\text{NH}}_{4}^{+}$ was positively correlated with Zn in fruit. For microelements, ACa, AMg, AFe, AMn, AZn, and ACu were markedly related to several mineral elements in the fruit (Figure 5).

The molecular ecological networks exhibited remarkable modular features with modularity of 0.634, dividing the microbes into nine modules (Figure 6). The relative abundances of microbes in modules 0, 1, 2, 3, and 5 were notably correlated with specific fruit quality indicators (Figure 6). For instance, the relative abundances of microbes in module 5 were prominently and positively correlated with B, Cu, and Ni, and negatively correlated with Mn, Zn, and total flavones in the fruit (Figure 6). Additionally, the relative abundances of microbes in module 2 were negatively correlated with B, Ca, Mg, Mn, and Ni, and positively correlated with Zn in the fruit. Module 5 contained seven bacterial genera and one protist genus: *Edaphobaculum, Devosia, Ferruginibacter, Variovorax, Luteimonas, Brevundimonas, Oligoflexus*, and *Gonostomum* (protist; Supplementary Table 4). Module 2 contained the following 18 bacterial genera: *Chujaibacter, Acidipila, Rhodanobacter, Granulicella, Candidatus_Udaeobacter, Mycobacterium, Sporosarcina, Bdellovibrio, Tumebacillus, Acidicaldus, Acidisoma, Candidimonas, Bordetella, Nitrolancea, Arachidicoccus, Mizugakiibacter, Acidiphilium*, and *Pedomicrobium* (Supplementary Table 4).

## Discussion

The significance of microelements in regulating microbial communities in marine ecosystems (Xu et al., 2021) and animal digestive systems (Littlejohn et al., 2023) gaining increasing recognition. However, their contribution of microelements to explaining microbial structure and function in terrestrial ecosystems remains largely understudied (Shepherd and Oliverio, 2024). In this study, microelements emerged as more influential in shaping the microbial community structure compared to geographical position, basic soil properties, and macroelements. This finding is consistent with previous research Dai et al. (2023), underscoring the importance of microelements in soil microbial communities. However, while previous studies have emphasized that AFe is the primary factor in determining microbial communities among microelements, followed by ACu (Dai et al., 2023). In this study, AFe significantly affected microbial communities but was not the primary factor; AMg was the most important factor, followed by ACu. This heterogeneity may have resulted from the differences in the scale of the research or discrepancies in soil type. Peng et al. (2022) and Dai et al. (2023) mainly focused on microbial communities at the continental scale, whereas we focused on local areas in Ninghai County, Zhejiang Province, China. The soil type used in our study was Ultisol, rich in Fe and Al, potentially explaining why iron may not have been a limiting factor for the microbiome. Mg plays a crucial role in the growth, metabolism, and reproduction of microorganisms (Wang et al., 2019). However, in most soils, the majority of Mg is incorporated into minerals and remains inaccessible to plants or microorganisms (Senbayram et al., 2015). The depletion of Mg in acidic soils, as observed in our study with soil pH ranging from 4.2 to 5.4, is exacerbated due to high saturation of the soil cation exchange capacity with H^+^ ions, leading to Mg leaching. The short-term application of magnesium fertilizer can influence the activity and composition of soil microbial communities (Gransee and Führs, 2013). Overall, our results suggest that the effects of microelements on microorganisms may vary depending on scale or soil type. At the same time, we have to recognize that still a large part of soil microbial community variation could not be explained by the soil physi-chemical properties we measured. In fact, although we have measured numerous indicators, there are still a great many soil properties that affect the soil microbial community, such as texture and structure, organic carbon quality, sulfur, and plant diversity (Fierer, 2017; Yin et al., 2023), were not determinated in this study, due to limited experimental conditions. Additionally, in our study, microbes exhibited significant phylogenetic signals for all microelements, and as soil microelements diverged, the dominant community assembly process shifted from homogeneous to stochastic and then to heterogeneous selection. To our knowledge, this is the first study to underscore the important role of micronutrients in soil community assembly. Previous studies often overlooked micronutrients, considering them secondary to soil basic properties and macroelements (Zhang et al., 2023; Zhong et al., 2023).

Presently, more and more studies revealed that soil microorganisms have the potential to influence fruit quality and quality. For example, the rhizospheric and endophytic microorganisms were reported to promote monoterpenes production in citrus (Su et al., 2023). In addition, *Bacillus* and *Mortierella* were proved to be positively correlated with grape yield and aroma compound contents in a continuous cropping grape orchard (Li et al., 2024). *Burkholderia* was effectively used as biocontrol agents to reduce fungal diseases and could promote the kiwifruit yield and quality (Liu et al., 2024). Moreover, in our previous study, we found that the richness of soil bacteria and protists was positively correlated with the average fruit weight of the loquat tree (Wang et al., 2024). However, there is still few studies have focused on the relationship between soil microbial diversity and fruit mineral nutrition. In this study, we found that microelements and the microbiome emerged as the top two essential factors that individually explaining 11.0 and 11.4% of fruit quality variation, respectively. Our network analysis of soil abiotic and biotic properties and fruit quality revealed that microelements and microbes mainly influenced the mineral elemental contents of loquats. Fe, Zn, Cu, Co, Mo, and Ca are trace elements essential for human health (Nieder et al., 2018). While, Zn is a component of many enzymes in the body, and Zn deficiency can cause taste dysfunction, stunted growth, skin damage, and immune function damage (Chasapis et al., 2020). A Cu deficiency may result in microcellular hypochromic anemia (Bost et al., 2016). In our study, the Zn content in loquats was positively correlated with the soil ${\text{NH}}_{4}^{+}$ and AMg and negatively correlated with soil ACu. The Zn content in loquats was positively correlated with soil pH and AMg, and Ca was positively correlated with soil AZn. These results emphasize the importance of adding Mg and Zn fertilizers to acidic loquat orchard soils to improve the nutritional value of fruits. Microbial fertilizers hold promise because they are environmentally friendly and can improve crop quality and yields (Jansson et al., 2023; Poppeliers et al., 2023). Additionally, we discovered that several microbial alliances affected the mineral contents of loquat fruits. These results offer initial clues for the development of microbial fertilizers that can precisely control the beneficial mineral elements in fruit. However, the relationship between fruit nutrients and the soil microbiome is complex, and further experiments that combine microbial culturomics, synthetic microflora, and other technologies are required to verify the effects of microbial alliances on the mineral element contents of loquat fruits (Trivedi et al., 2020; Jansson et al., 2023).

**Figure 7:**
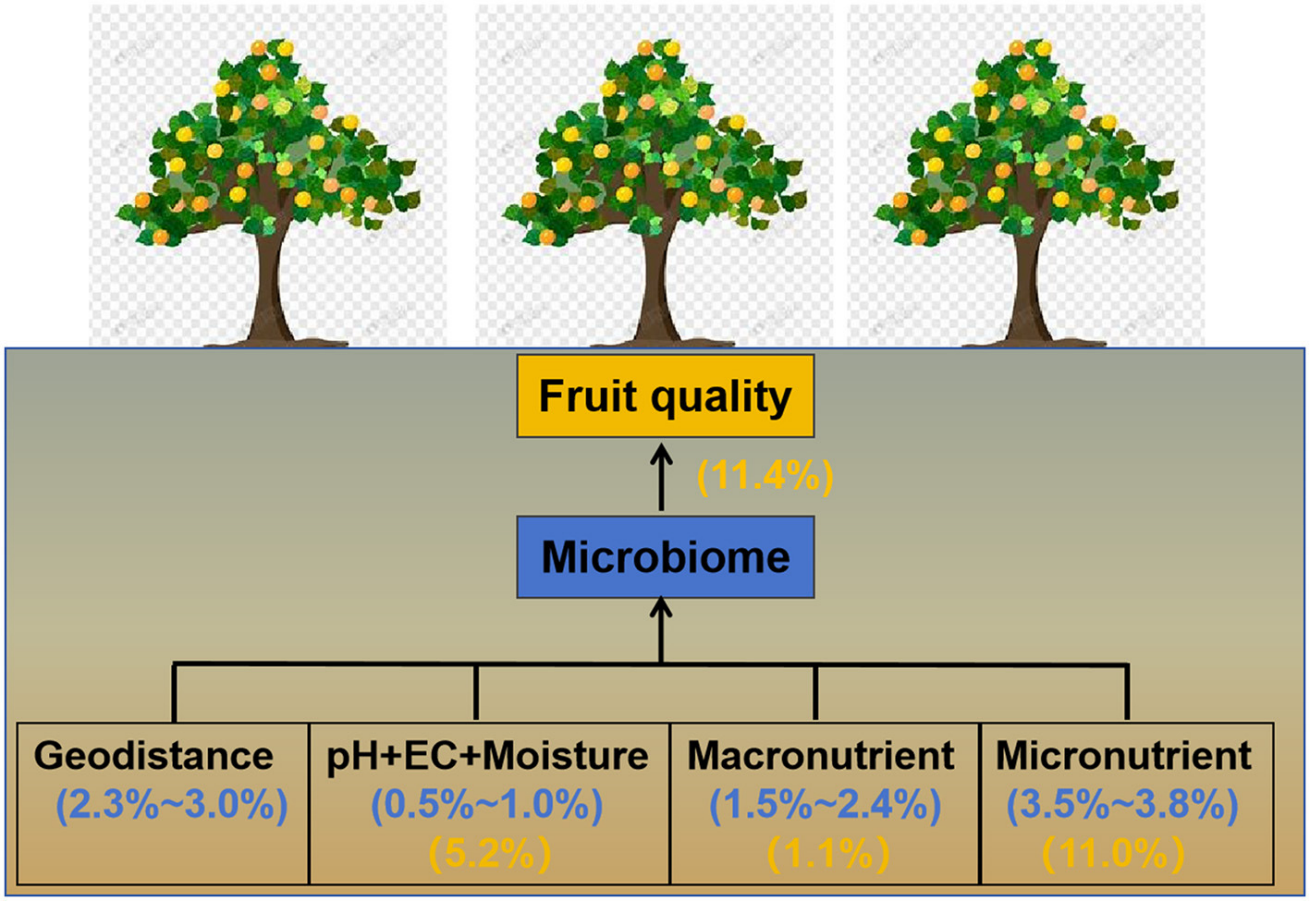
Conceptual diagram illustrating the effects of geodistance, basic soil properties (pH+EC+moisture), soil micronutrients, and soil macronutrients on the soil microbiome and fruit quality. The numbers in parentheses represent the individual contributions of soil properties to the soil microbiome (blue) and fruit quality (yellow), as estimated via variance decomposition analysis.

Our study unveiled a more prominent role of micronutrients in the soil microbiome compared to geographic position (longitude, latitude, and elevation), basic soil properties (pH+EC+moisture), and macroelements (C, N, and P), which have generally been overlooked in previous studies (Figure 7). AMg and ACu emerged as the two most influential microelements shaping the soil microbiome. Concerning fruit quality, the mineral nutritional contents of the fruit were primarily influenced by microelements and the soil microbiome. These findings underscore the significance of microelement fertilization in orchard management. Moving forward, further research is warranted to delve into the ecological functions of microelements, such as their impact on soil multifunctionality, soil health index, and soil element cycling.

## Data availability statement

Our DNA sequencing data was deposited to the China National Center for Bioinformation (CNCB) database (https://www.cncb.ac.cn/) under BioProject accession number PRJCA021566.

## Author contributions

XW: Formal analysis, Investigation, Methodology, Project administration, Writing – original draft, Writing – review & editing. LW: Project administration, Supervision, Writing – original draft, Writing – review & editing. BW: Data curation, Resources, Writing – review & editing. ZY: Data curation, Formal analysis, Funding acquisition, Writing – review & editing. YZ: Investigation, Methodology, Resources, Writing – review & editing. LQ: Methodology, Resources, Software, Writing – review & editing. MW: Data curation, Methodology, Resources, Validation, Visualization, Writing – review & editing. YW: Methodology, Resources, Writing – review & editing. TG: Funding acquisition, Project administration, Resources, Supervision, Writing – review & editing. ZZ: Project administration, Resources, Writing – review & editing.
