# Supplementary material for: Neglected role of microelements in determining soil microbial communities and fruit micronutrients in loquat orchards
Source: Front Microbiol. 2024 Aug 21;15:1447921. doi: 10.3389/fmicb.2024.1447921 (PMC11373571; doi:10.3389/fmicb.2024.1447921)
Supplement: Supplementary file 1 [file Data_Sheet_1.pdf]

Supplementary materials

Neglected role of microelements in determining soil microbial communities and fruit quality in loquat orchards

Materials and methods

Fruit quality parameters

Fruit weights were measured using a 0.001 g confidence level precision balance. Soluble solids in fruit juice were measured using a hand refractometer. An ascorbic acid assay kit (Merck, Darmstadt, Germany) based on a colorimetric method was used to detect vitamin C contents. Total flavone content was measured using aluminum ion colorimetry (Shraim *et al.*, 2021). Dietary fiber and reducing sugar contents were determined following the methods recommended by the official AOAC method for fruits (Powell Gaines, 1973). The contents of various mineral elements, including K, Na, Ca, Mg, Fe, Zn, Cu, Co, Mo, Mn, Pb, B, Ba, Tl, Li, and Ni were measured using an inductively coupled plasma optical emission spectrometer (ICP-OES), model ICAP 6300 Duo, Thermo-Fisher Scientific (Waltham, MA, USA) with an auto sampler (CETAX model ASX-520) (Khan *et al.*, 2022).

Soil physicochemical properties

Dissolved organic carbon (DOC) in the soils was determined using a TOC/TN analyzer (Tekmar Dohrmann Apollo 9000). Dissolved organic nitrogen (DON), nitrate (NO_3^-), and ammonia (NH_4^+) were extracted using 2 M KCl and analyzed using a FIAstar 5000 analyzer (Foss Tecator, Denmark) (Wang *et al.*, 2018). Total phosphorus (TP) was estimated using vanadium molybdate yellow colorimetry (Nelson and

Sommers, 1983). Available phosphorus (AP) was measured as described by Olsen et al. (Olsen, 1954). The total potassium (TK) was determined using the HF and $HClO_4$ digestion methods (Jackson, 1958). Available potassium (AK) was determined using flame-emission photometry (Okalebo *et al.*, 2002). Soil pH and electrical conductivity (EC) were measured using pH and conductivity meters, respectively. The gravimetric water content (i.e., soil moisture) was determined by drying fresh soil in an oven at 105 °C for 48 h.

References

Jackson M. Soil chemical analysis prentice Hall. Inc, Englewood Cliffs, NJ. 1958;498:183-204.

Khan, S.R., Sharma, B., Chawla, P.A., Bhatia, R., 2022. Inductively coupled plasma optical emission spectrometry (ICP-OES): a powerful analytical technique for elemental analysis. Food Analytical Methods, 1-23.

Nelson Da, Sommers LE. Total carbon, organic carbon, and organic matter. Methods of soil analysis: Part 2 chemical and microbiological properties. 1983;9:539-79.

Okalebo JR, Gathua KW, Woomer PL. Laboratory methods of soil and plant analysis: a working manual second edition. Sacred Africa, Nairobi. 2002;21:25-6.

Olsen SR. Estimation of available phosphorus in soils by extraction with sodium bicarbonate: US Department of Agriculture; 1954.

Powell Gaines T. Automated determination of reducing sugars, total sugars, and starch in plant tissue from one weighed sample. Journal of the Association of Official Analytical Chemists. 1973;56(6):1419-24.

Shraim AM, Ahmed TA, Rahman MM, Hijji YM. Determination of total flavonoid content by aluminum chloride assay: A critical evaluation. Lwt.

2021;150:111932.

Wang L, Luo X, Liao H, Chen W, Wei D, Cai P, et al. Ureolytic microbial community is modulated by fertilization regimes and particle-size fractions in a Black soil of Northeastern China. Soil Biology and Biochemistry. 2018;116:171-8.

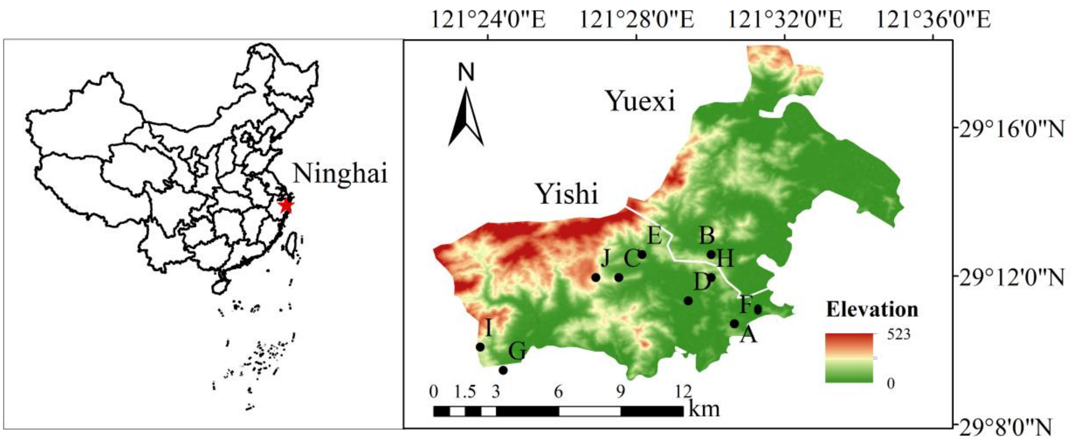

Fig. S1 Sampling site map.

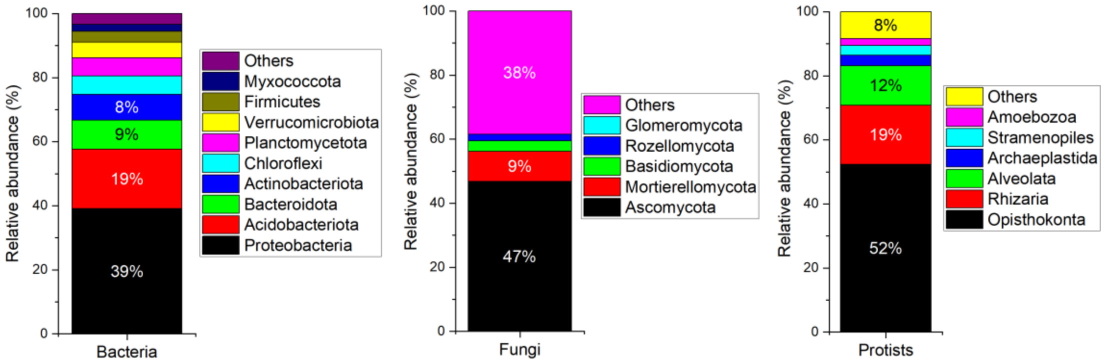

Fig. S2 The relative abundances of major microbial taxa.

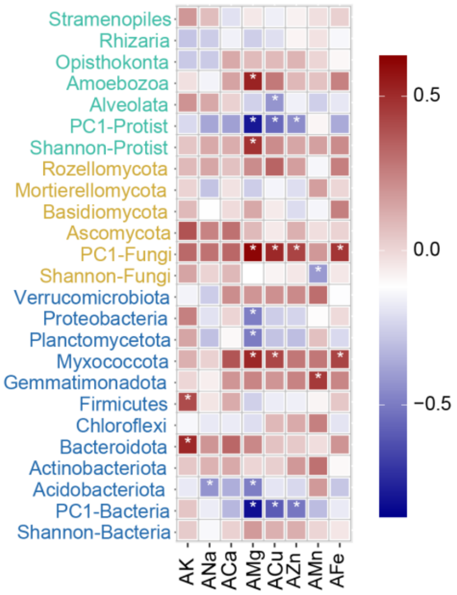

Fig. S3 Spearman's correlations between micronutrients and microbial community structure (PC1), diversity (Shannon), and relative abundances of major microbial taxa.

Table S1 Basic information of the 10 studied loquat orchards.

ID	Location	Longitude (°E)	Latitude (°N)	Cultivation time (year)	Fertilization[‡]	Soil pH [‡]	Soil EC[§] (mS/cm)
1	A	121.51	29.18	<10 a	IO	4.9	253.0
2	B	121.50	29.21	<10 a	IO	5.2	158.1
3	C	121.46	29.20	<10 a	I	5.0	82.9
4	D	121.49	29.19	10–15 a	I	4.2	275.1
5	E	121.47	29.21	10–15 a	O	4.3	263.3
6	F	121.52	29.19	15–20 a	I	4.7	110.4
7	G	121.41	29.16	15–20 a	IO	4.9	315.1
8	H	121.50	29.20	15–20 a	IO	5.4	207.6
9	I	121.40	29.17	>20 a	I	5.4	72.8
10	J	121.45	29.20	>20 a	O	4.4	137.7

[‡] I represents inorganic fertilizer as a local practice, O represents organic fertilizer as a

local practice, and IO represents inorganic fertilizer plus organic fertilizer.

‡ The average soil pH of the loquat orchards.

§ The average soil EC of the loquat orchards.

Table S2 Primers used to determine soil bacterial, fungal, and protistan communities.

Microbes	Primer name	Primer sequences	Reference
Bacteria	515F<br>907R	GTGCCAGCMGCCGCGG<br>CCGTCAATTCMTTTRAGTTT	Jiao *et al.*, 2022
Fungi	ITS5-1737F<br>ITS2-2043R	GGAAGTAAAAGTCGTAACA AGG<br>GCTGCGTTCTTCATCGATGC	Jiao *et al.*, 2022
Protists	TAReuk454FWD1<br>TAReukREV3	CCAGCASCYGCGGTAATTCC<br>ACTTTCGTTCTTGATYRA	Du *et al.*, 2022

Table S3 Mantel test between environmental properties and soil microbial community structure.

	Property	Bacteria	Fungi	Protists
Geographical properties	Latitude	0.103	0.161**	0.135*
	Longitude	0.156*	0.177**	0.182**
	Altitude	0.170**	0.059	0.077
Physicochemical properties	pH	0.334***	0.279***	0.338***
	EC	0.119*	-0.241**	0.133*
	Moisture	0.078	0.167**	0.181**
Macroelements	SOC	0.050	0.089	0.021
	DOC	0.033	0.073	0.044
	DON	0.281***	0.165**	-0.209***
	NO3-	0.152*	0.172**	0.181**
	NH4+	0.108	0.091	0.064

	AP	0.174**	0.203**	0.235***
Microelements	AK	0.252***	0.174**	-0.237***
	ACa	0.124*	0.101	0.144*
	ANa	0.161**	0.079	0.129*
	AMg	0.384***	0.346***	0.361***
	AFe	0.180**	0.130*	0.109
	AMn	0320***	0.161**	0.189**
	AZn	0.295***	0.308***	0.289***
	ACu	0.354***	0.316***	0.355***

Note: The abbreviations for the environmental factors are defined in the Methods and Materials section. Asterisks indicate statistical significance.

* $P < 0.05$.

** $P < 0.01$.

*** $P < 0.001$.
